# Differences across health care systems in outcome and cost-utility of surgical and conservative treatment of chronic low back pain: a study protocol

**DOI:** 10.1186/1471-2474-9-81

**Published:** 2008-06-06

**Authors:** Markus Melloh, Christoph Röder, Achim Elfering, Jean-Claude Theis, Urs Müller, Lukas P Staub, Emin Aghayev, Thomas Zweig, Thomas Barz, Thomas Kohlmann, Simon Wieser, Peter Jüni, Marcel Zwahlen

**Affiliations:** 1 MEM Research Center for Orthopaedic Surgery, University of Berne, Stauffacherstrasse 78, 3014 Berne, Switzerland; 2 Section of Orthopaedic Surgery, Department of Medical and Surgical Sciences, Dunedin School of Medicine, University of Otago, Private Bag 1921, Dunedin, New Zealand; 3 Department for Work and Organizational Psychology, Institute for Psychology, University of Berne, Muesmattstrasse 45, 3000 Berne 9, Switzerland; 4 Department of Orthopaedic Surgery, Asklepius Klinikum Uckermark, Auguststrasse 23, 16303 Schwedt/Oder, Germany; 5 Department of Methods in Community Medicine, Institute for Community Medicine, University of Greifswald, Walther-Rathenau-Strasse 48, 17487 Greifswald, Germany; 6 Institute of Health Economics, Zurich University of Applied Sciences, Im Park, St. Georgenstrasse 70, Postfach 958, 8401 Winterthur, Switzerland; 7 Research Support Unit, Institute of Social and Preventive Medicine (ISPM), University of Berne, Finkenhubelweg 11, 3012 Berne, Switzerland

## Abstract

**Background:**

There is little evidence on differences across health care systems in choice and outcome of the treatment of chronic low back pain (CLBP) with spinal surgery and conservative treatment as the main options. At least six randomised controlled trials comparing these two options have been performed; they show conflicting results without clear-cut evidence for superior effectiveness of any of the evaluated interventions and could not address whether treatment effect varied across patient subgroups. Cost-utility analyses display inconsistent results when comparing surgical and conservative treatment of CLBP. Due to its higher feasibility, we chose to conduct a prospective observational cohort study.

**Methods:**

This study aims to examine if

1. Differences across health care systems result in different treatment outcomes of surgical and conservative treatment of CLBP

2. Patient characteristics (work-related, psychological factors, etc.) and co-interventions (physiotherapy, cognitive behavioural therapy, return-to-work programs, etc.) modify the outcome of treatment for CLBP

3. Cost-utility in terms of quality-adjusted life years differs between surgical and conservative treatment of CLBP.

This study will recruit 1000 patients from orthopaedic spine units, rehabilitation centres, and pain clinics in Switzerland and New Zealand. Effectiveness will be measured by the Oswestry Disability Index (ODI) at baseline and after six months. The change in ODI will be the primary endpoint of this study.

Multiple linear regression models will be used, with the change in ODI from baseline to six months as the dependent variable and the type of health care system, type of treatment, patient characteristics, and co-interventions as independent variables. Interactions will be incorporated between type of treatment and different co-interventions and patient characteristics. Cost-utility will be measured with an index based on EQol-5D in combination with cost data.

**Conclusion:**

This study will provide evidence if differences across health care systems in the outcome of treatment of CLBP exist. It will classify patients with CLBP into different clinical subgroups and help to identify specific target groups who might benefit from specific surgical or conservative interventions. Furthermore, cost-utility differences will be identified for different groups of patients with CLBP. Main results of this study should be replicated in future studies on CLBP.

## Background

Acute low back pain (ALBP) affects more than 70% of all people at least once in their lifetime [1], and about 5% develop nonspecific CLBP, which is defined as LBP lasting longer than 12 weeks [2]. Although this is only a small group of patients, the economic costs exceed those for the treatment of ALBP by a significant amount [3].

Guidelines already exist for the management of CLBP [4], but the main question in CLBP–whether to offer spinal surgery or conservative treatment–is not clearly answered [5].

With both intervention rates in spinal surgery and health care consumers' expectations rising, critics are voicing concern about a possible "overuse" of spinal surgery [6]. Conservative treatment of low back pain is an alternative that might bridge the self-healing processes [7]. Some authors see advantages for surgical procedures in terms of cost-utility [8], whereas others see no significant differences between both treatment groups [9]. There is weak evidence on differences across health care systems concerning outcomes of CLBP that favours countries with a National Health System (NHS) compared to countries with a private health care system [10,11]. Evidence and outcome-based research is needed to provide information on the appropriate indication for spinal surgery [12]. The aim would be to identify specific target groups that will benefit from specific interventions and classify patients with CLBP into different clinical subgroups [13].

At least six randomised trials have been performed comparing the outcomes of spinal surgery and nonsurgical treatment in CLBP–most of them with some problems in conduct.

Among these are the study by the Swedish lumbar spine study group comparing patients with spinal surgery and patients with continuing physical therapy [14]; the Norwegian spine study comparing patients with lumbar fusion plus physiotherapy, and patients with cognitive intervention plus exercises [15]; the UK Medical Research Council trial comparing patients with spinal stabilisation and patients with an intensive rehabilitation programme [16]; and the American Spine Patient Outcomes Research Trial (SPORT) comparing patients with discectomy and patients with physical therapy plus exercise therapy [17]. Further RCTs have been conducted for spondylolisthesis. Some RCTs had to be closed because patients did not want surgery.

These trials showed conflicting results, with no treatment being clearly superior [18]. Exclusion criteria in all these trials were restrictive; for example, patients with mild CLBP and no need for surgery, as well as patients with progressive neurological abnormalities and surgery were excluded. Recruitment in all trials was long and varied between four and six years. This leads to a reduced generalizability of the results.

The analysis of data from an observational cohort with adequate statistical methods can lead to results with an evidence level 2++. It is slowly recognized that for certain clinical questions RCTs have limitations [19] and may be unnecessary, inappropriate, or impossible to conduct [20].

Feasibility is an important aspect and has been highlighted by the SPORT trial, which consists of a randomised and an observational cohort. The rate of surgical patients with treatment crossover within two years of treatment allocation was 40% in the RCT, compared to merely 4% in the observational cohort [17,21]. The comparable rates of conservative patients' crossover to surgery were 45% and 22%. Currently, a prospective cohort study comparing patients with lumbar fusion and patients with multidisciplinary therapy is under way, for which a randomised cohort design was planned, but was changed to an observational design to minimise a treatment crossover [22].

Therefore, we chose to conduct a prospective observational cohort study as well, with a lower level of evidence than an RCT though, but a higher feasibility.

## Methods/design

### Research plan

#### Objectives and goals

This study aims to identify differences across health care systems in the Swiss and New Zealand populations such as different compensation schemes, patient characteristics, and co-interventions that modify the effects of surgical and conservative treatment of patients with CLBP. In addition, differences in cost-utility will be identified for different groups of patients.

#### Hypotheses

Three hypotheses will be examined:

1. Differences across health care systems result in better treatment outcomes of surgical and conservative treatment of CLBP in countries with a NHS.

2. Patient characteristics, such as high job satisfaction and short pain duration, and co-interventions, such as cognitive behavioural therapy and return-to-work programs, increase the effectiveness of surgical and conservative treatment for CLBP.

3. Cost-utility in terms of quality-adjusted life years (QALYs) is higher in conservative than in surgical treatment of CLBP.

Previous inconclusive findings may have resulted from potential effect modifiers that varied across different health care systems. For instance, expectations to return quickly to work may function as an effect modifier depending on the health compensation system. This study will examine whether the results pertaining to hypotheses 2 and 3 vary by health care system.

#### Study design

In this multinational prospective cohort study, patients will be recruited consecutively from orthopaedic spine units, rehabilitation centres, and pain clinics in Switzerland (CH) and New Zealand (NZ). Participants will be assessed pre-intervention and at six-month follow-up. The duration of treatment will be a maximum of six months per patient.

The study protocol has been approved by the Ethics Commission of the Canton of Berne (KEK-Gesuchs-Nr.: 191/07).

#### Inclusion and exclusion criteria

We defined broad inclusion criteria to ensure that the spectrum of patients represents the spectrum seen in routine settings: CLBP with a duration of the current episode of at least three months, no relevant improvement under prior conservative treatment for one year, patients about to undergo surgical or further conservative treatment, age 18–65 years at beginning of treatment, good understanding of German in the Swiss patient sample or English the New Zealand sample, and written consent (see table 1).

**Table 1:** Inclusion and exclusion criteria

1. CLBP	7. specific CLBP
2. no relevant improvement under prior conservative treatment for one year	8. acute or subacute LBP
3. about to undergo surgical or further conservative treatment	9. comorbidity that determines overall well-being
4. age 18–65 years at the beginning of treatment	10. pregnancy
5. good understanding of German (CH) or English (NZ)	11. expected loss to follow-up
6. written consent	12. unwillingness to complete questionnaires

Exclusion criteria are specific CLBP (as infection, tumour, osteoporosis, ankylosing spondylitis, fracture, deformity, inflammatory process, and cauda equina syndrome), acute or subacute LBP (defined as LBP continuing for less than six respectively twelve weeks at beginning of treatment [1,4], comorbidity that determines overall well-being (e.g. painful/disabling arthritic hip joints), pregnancy, expected loss to follow-up (e.g. due to removal), and unwillingness to complete questionnaires (see table 1).

### Interventions

#### Surgical treatment

Surgical interventions for CLBP in this study are fusion (anterior and posterior lumbar interbody fusion, posterolateral instrumented fusion) and nonfusion surgery (decompression, dynamic stabilization, total disc replacement). Given the nature of interventions, a sham intervention is not feasible.

#### Conservative treatment and co-interventions

We defined the absence of a surgical treatment as conservative treatment. Different conservative treatments are considered as co-interventions. These co-interventions that also can be applied to the surgical patient group are biomedical, psychologically oriented (cognitive behavioural therapy), and socio-occupational interventions (return-to-work program). Biomedical interventions include physical treatments, exercise therapy, manual therapy, back schools, pharmacological procedures (analgetics, NSAID, opioids, antidepressants), and invasive nonsurgical treatments (acupuncture, injections, facet/nerve root blocks, neuroreflexotherapy, percutaneous electrical nerve stimulation, radiofrequency, intradiscal electrothermal therapy, intradiscal radiofrequency thermocoagulation, cryotherapy of the facets, alcohol denervation, temporary epidural catheters, permanent intrathecal pain-control pumps, and spinal cord stimulators).

### Outcome assessment

#### Demographic and baseline characteristics

The following demographic and baseline characteristics will be collected by patient self-assessment: gender, age, comorbidity (SCQ), marital/relationship status, smoking behaviour, onset/duration/recurrence of LBP, number/level of previous spine surgeries, duration of previous conservative treatment, educational/work/disability compensation status, work history, biomechanical workload, duration of work-absenteeism, job satisfaction, work stress factors, job control, social recognition of LBP from supervisors and colleagues, pain tolerance during work, belief that work has caused LBP, recovery/emotional distress after work, social stress factors, search for social support, condition-specific disability (ODI), general physical and mental health (SF-36), cost-utility of treatment (index based on EQol-5D in combination with cost data), intensity of back and leg pain (VAS), pain frequency, location and number of other painful body sites (SEQ pain assessment), pain behaviour, pain control, self-efficacy, family reinforcement of pain, interference of LBP with work and/or activity, symptom-specific well-being, general well-being, somatisation (MSPQ), depression (ZUNG), fear avoidance beliefs (FABQ), coping strategies (CSQ), and patients' expectations.

Physician-based assessment of patient characteristics at baseline include centre/clinic of treatment, in/outpatient treatment, height, weight, and medication.

#### Outcome measures

Relevant outcomes are identified as scores organised in categories of answers in standardised multi-dimensional assessment tools. Significance of outcomes is defined by effect sizes (i.e. differences between treatment groups in changes from baseline to endpoint, typically divided into three groups: short-, medium-, and long-term changes of respective scores). For this study, outcome measures will be assessed at six-month follow-up. Longer follow-ups may result in difficulties for the identification of effect modifiers [23].

ODI [24,25], SF-36 [26], EQol-5D [27], and pain measured by VAS [28] are among the most frequently used outcome assessment measurements of CLBP. All of them are valid and reliable. In this study, the primary endpoint at six-month follow-up is ODI. Key secondary endpoints are SF-36, EQol-5D, and VAS.

Other secondary outcomes that will be collected by patient self-assessment are onset/duration/recurrence of LBP [29-31], work/disability compensation status [32,33], duration of work-absenteeism [34], location and number of other painful body sites [35,36], interference of LBP with work and/or activity, symptom-specific well-being, general well-being, and satisfaction with treatment.

Physician-based assessment of secondary treatment outcomes at six-month follow-up include rate/type/therapeutic consequences of complications [37], change in medication, and overall outcome (McNab criteria).

Given the nature of interventions, blinding is not feasible. Outcome assessments are based on standardised, reliable, valid tools.

#### Effect modification

Possible effect modifiers are all co-interventions, as well as all demographic and baseline characteristics [23,38-40], which include gender [41], age [42], severity of pain [43], comorbidity [44], socioeconomic status [45], work-related factors [34,46,47], indirect reward parameters due to the health care system [48], psychosocial distress [49-52], somatisation and depression [53,54], fear-avoidance beliefs and coping strategies [55,56], and expectations [57].

#### Cost-utility

The socioeconomic objective of the study is to evaluate the cost-utility outcomes of surgical and conservative treatment of CLBP [8,9,27]. The costs considered include the direct health care expenditures borne by the health care system (insurance, public health care) and out-of-pocket expenditures by patients (medication, alternative medicine, personal prevention activities). The indirect social costs due to lost productivity because of absenteeism also will be accounted for [58]. Direct medical costs will collected from health insurance companies in CH, the NHS in NZ and the clinics in which the patients are selected. Indirect costs and out-of-pocket costs will be measured with a cost diary developed by the Zurich University of Applied Sciences in cooperation with the MEM Research Center. The cost diary will be kept by the patients on a weekly basis covering the total period of treatment with a maximum of six months. At beginning of treatment and end of follow-up period utility will be measured with an index based on EQol-5D and SF-36. Changes in the index will be translated into changes in QALYs.

The cost study of CLBP patients will concentrate on the segment of high-cost patients and address this question: Is this group homogeneous with respect to their socioeconomic status, care-seeking and back pain patterns? If the answer is no, are there subgroups whose cost may be reduced substantially by appropriate (nonmedical) support?

The cost-utility part of the study will be done in collaboration with the Zurich University of Applied Sciences, Institute of Health Economics.

### Data management

#### Database

The backbone of this Swiss-based study is the IT-infrastructure of the MEM Research Center, University of Berne, which has developed and improved documentation systems and questionnaires over decades.

Orthopaedic spinal units, rehabilitation centres, and pain clinics in CH and NZ will participate in this study. Collaborators in the main clinics will act as local coordinators in association with local research nurses. The outcome assessment is based on physician- and patient-derived data.

After written informed consent patients will be examined by the surgeons or physicians under whose care they are. Research nurses will hand out self-assessment questionnaires to the patients and will be available to help completing these questionnaires if necessary. The completed patient-based questionnaires will be collected by the research nurses and, together with physician-based questionnaires, forwarded to the MEM Research Center in CH respectively to the University of Otago in NZ. All data will be saved at the central database of the main server at the University of Berne.

#### Data security

The computerised medical data collected at the MEM Research Center do not contain names. Only the patient number will be able to connect the names and the medical information at the treatment level. The servers have firewalls and data encryption, and are located in a physically secure environment.

### Statistical Analysis

#### Outcomes

In this prospective cohort study, two interventions for CLBP are compared: surgical and conservative treatment. Subgroups of different surgical treatments can be distinguished. Nonsurgical co-interventions may have an effect on patient groups with surgical as well as with conservative treatment.

Follow-up data at six months will be analysed according to the intention-to-treat principle, except for those patients who change treatment groups before the beginning of the allocated treatment.

Changes in dependent outcome variables between baseline and follow-up at six months will be analysed by multiple linear regression models. Surgical and conservative treatment, patient characteristics, and co-interventions are independent variables. To address potential effect modification, we will then incorporate interactions between type of treatment and the different co-interventions and patient characteristics.

Highest priority among co-interventions will be given to the analysis of effects of cognitive behavioural therapy and return-to-work programs. Among patient characteristics highest priority in data analysis will be given to gender, age, and duration of work-absenteeism.

#### Costs

The most important cost predictors (including cost drivers) for total, direct, and indirect costs are identified by multivariate modelling approaches.

One multivariate modelling approach is to determine which individuals with CLBP are in the top, say, 15% of the total cost, and to contrast this group with the remaining individuals. The relevant cost predictors are identified by discriminant analysis, or any other suitable classification method such as logistic classification or classification trees.

Another approach is to model the costs by a multiple regression type model, where the costs are assumed to be log-Gaussian or gamma distributed. The uncertainty in the prediction of cost will be quantified by state-of-the-art methods [59,60].

### Sample size/power calculation

Sample size considerations in clinical studies start from what is considered a minimal clinically important difference. In studies of chronic low back pain, the ODI is commonly used as the main outcome measure, and a difference in 10 ODI points is considered clinically relevant [61,62]. This study, however, aims at investigating effect modification (by several candidate variables) of the treatment effect of surgical versus conservative treatment. This makes higher sample sizes necessary to have adequate power to answer the question [63,64].

We considered a series of scenarios for sample size calculations. To simplify the scenarios, we assumed a 1:1 distribution between patients undergoing surgical and conservative treatment, and a 1:1 distribution for the candidate effect modifier variable (e.g., gender).

We then made assumptions about the standard deviation of changes in ODI over a six-month period. From published studies and unpublished data sets we calculated scenarios with standard deviation ranging from 18 to 23 [14,16,21].

In general, an interaction effect of 0.4 to 0.6 of the main treatment effect is considered relevant.

These assumptions resulted in an array of sample sizes between 500 to 800 necessary to ensure 80% power to assess the hypothesized levels of effect modifications.

Furthermore, we assumed a 20% rate of patients who will not return on schedule to the six-month clinical visit at which the main outcome measurements will be conducted. These calculations showed that at least 800 patients should be included to provide 80% power to detect this interaction effect at a two-sided p of 0.05 [63]. However, as there is uncertainty about the distribution of effect modifiers, the proportion of patients opting for surgery, and the proportion that will not be seen at six months, we decided to set a target sample size of 1000 patients.

### Feasibility of the study

The patient sample will be taken from orthopaedic spine units, rehabilitation centres, and pain clinics in CH and NZ. The feasibility of data collection in CH has been proved in prior joint ventures between the MEM Research Center and university and regional hospitals (e.g. Inselspital and Salem-Spital in Berne, Schulthess Clinic in Zurich, all of them offering surgical and conservative treatment, none of them working on a referral basis).

NZ has been chosen for comparison of differences across health care systems because of ideal starting conditions for this research project: NZ is a country with a traditionally high acceptance of clinical research. For example, the National Joint Register of patients who underwent total hip or knee replacement, established in 1999, has near 100% coverage.

The MEM Research Center in Berne coordinates the Swiss-based European Spine Registry of the Spine Society of Europe (SSE) [65-70]. In parallel to the planned bi-national multicentre study, a national spine registry for NZ could be implemented by the University of Otago. This National Spine Register – comparable to the existing National Joint Register – would address all patients with CLBP who report to a clinic in NZ, generating synergetic effects. All patients joining the National Spine Register could be asked to participate in the multicentre study as well, which would make data collection much easier.

Cooperation on successful doctoral studies in both countries already exists between the University of Berne and the University of Otago, NZ.

Furthermore, NZ has a manageable population size, which is comparable to CH. This setting and the observational nature of the study will ensure sufficiently high recruitment rates.

**Figure 1:**
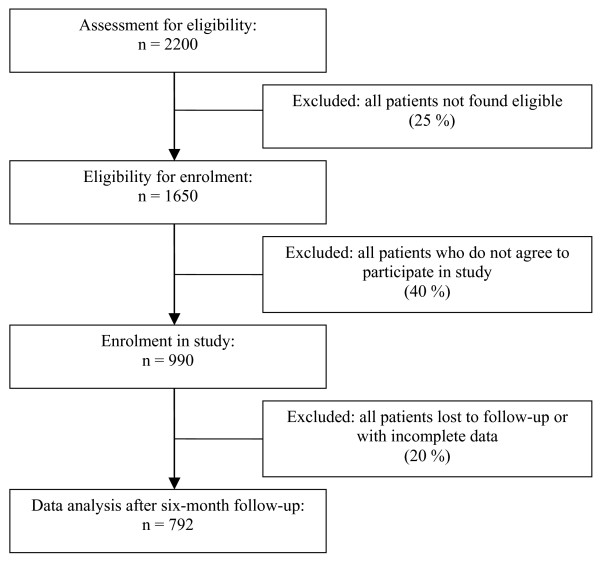
Recruitment and data collection.

The respective acquisition of patient data will be coordinated at the MEM Research Center and the University of Otago. 2200 patients will be assessed for eligibility in CH and NZ combined (see figure 1). It can be assumed that 75% of these screened patients will be found eligible for enrolment. Out of these 1650 patients, presumably 990 (60%) will agree to participate in this study. Up to 20% of the patients can be expected to be lost to follow-up in the worst case scenario, so that, finally, 792 patients will be analysed according to the intention-to-treat principle. This estimate is based on data provided by a recent prospective cohort study comparing surgical and conservative treatment in a LBP population, with ODI as primary outcome measures [21].

The expected duration of the entire study will be two and a half years, including two years of patient recruitment overlapping with two years of follow-up.

## Discussion

### Scientific significance

LBP is by far the most prevalent and most costly musculoskeletal problem in advanced economic societies today. Despite numerous previous studies, little is known about differences across health care systems and its predictors, and cost-utility analyses are rare. This study will provide evidence if differences in the treatment of CLBP exist across different health care systems. It will classify patients with CLBP into different clinical subgroups and help to identify specific target groups who will benefit from specific surgical or conservative interventions. These subgroups could be reassessed in future RCTs.

Findings from this study may lead to a paradigm shift in the treatment of patients with CLBP and moreover, may have an impact on future guidelines for the management of CLBP.

### Social and economic significance

We anticipate to identify cost-utility differences of treatment choices for different groups of patients with CLBP and to suggest areas with a high potential for improved cost effectiveness. Both health care providers and consumers might benefit if unnecessary spinal surgical procedures can be avoided.

## Abbreviations

ALBP, acute low back pain; CH, Switzerland; CLBP, chronic low back pain; CSQ, Coping strategies questionnaire; EQol-5D, European quality of life instrument measuring five dimensions; FABQ, Fear avoidance beliefs questionnaire; ISPM, Department of Social and Preventive Medicine; IT, information technology; LBP, low back pain; MEM, Maurice-E.-Mueller; MSPQ, Modified somatic perceptions questionnaire; NHS, National health system; NSAID, non-steroidal anti-inflammatory drug; NZ, New Zealand; ODI, Oswestry disability index; QALY, quality adjusted life year; RCT, randomised controlled trial; SCQ, Self-administered comorbidity questionnaire; SEQ, Standard evaluation questionnaire; SF-36, Short form 36 questionnaire; SPORT, Spine patient outcomes research trial; SSE, Spine Society of Europe; UK, United Kingdom; VAS, Visual analog scale; ZUNG, self-rating depression scale.

## Competing interests

The authors declare that they have no competing interests.

## Authors' contributions

MM is the principal investigator. He designed the study and is responsible for the protocol. CR and UM are the project leaders for CH and responsible for the involvement of clinics in CH. AE is the consultant on psychology who structured the ideas about psychosocial and work-related factors of LBP. J–CT is the project leader for NZ and responsible for the involvement of clinics in NZ. LPS is responsible for the development of the central database and documentation system. EA is responsible for the evaluation of the database. TZ contributed to the implementation of physician- and patient-based questionnaires, and to the data management. TB is the supervising spine surgeon of MM. TK is the sociological supervisor of MM. He structured the ideas about differences across health care systems in the treatment of LBP. SW is conducting design and data analysis on cost-utility. PJ contributed to the sample size/power calculation and to the design of statistical analysis. MZ is responsible for the sample size calculation and the design of statistical analysis. All authors participated in the study design as well as read and approved the final manuscript.

## Pre-publication history

The pre-publication history for this paper can be accessed here:


